# Ankyrin repeat and suppressor of cytokine signaling box containing protein-10 is associated with ubiquitin-mediated degradation pathways in trabecular meshwork cells

**Published:** 2013-07-25

**Authors:** Kate E. Keller, Yong-feng Yang, Ying Ying Sun, Renee Sykes, Ted S. Acott, Mary K. Wirtz

**Affiliations:** Casey Eye Institute, Oregon Health & Science University, 3181 SW Sam Jackson Park Rd, Portland, OR

## Abstract

**Purpose:**

Ankyrin repeat and suppressor of cytokine signaling (SOCS) box containing protein-10 (*ASB10*) was recently identified as a gene that causes primary open-angle glaucoma. Here, we investigated endogenous ASB10 protein expression in human trabecular meshwork (HTM) cells to provide the first clues to the biologic function of this protein.

**Methods:**

Primary HTM cells were cultured and immunostained with anti-ASB10 and various biomarkers of the ubiquitin-mediated proteasomal and autophagy-lysosomal degradation pathways. Cells were imaged with confocal and high-resolution structured illumination microscopy. Colocalization was quantified using Imaris Bitplane software, which generated a Pearson’s correlation coefficient value. Coimmunoprecipitation of ASB10-transfected cells was performed.

**Results:**

Immunofluorescence and confocal analysis showed that ASB10 was localized in intracellular structures in HTM cells. Two populations were observed: small, spherical vesicles and larger, less abundant structures. In the ASB10-silenced cells, the number of large structures was significantly decreased. ASB10 partially colocalized with biomarkers of the ubiquitin-mediated proteasomal pathway including ubiquitin and the α4 subunit of the 20S proteasome. However, ASB10 itself was not ubiquitinated. ASB10 also colocalized with numerous biomarkers of specific autophagic structures: aggresomes (histone deacetylase 6 [HDAC6] and heat shock protein 70 [HSP70]), autophagosomes (light chain 3 [LC3] and p62), amphisomes (Rab7), and lysosomes (*lysosomal-associated membrane protein 1 [*LAMP1]). Pearson coefficients indicated strong colocalization of large ASB10-stained structures with the α4 subunit of the 20S proteasome, K48 and K63-linked ubiquitin antibodies, p62, HSP70, and HDAC6 (Pearson’s range, 0.59–0.82). Coimmunoprecipitation assays showed a positive interaction of ASB10 with HSP70 and with the α4 subunit of the 20S proteasome. Super-resolution structured illumination confocal microscopy suggested that the smaller ASB10-stained vesicles aggregated into the larger structures, which resembled aggresome-like induced structures. Treatment of HTM cells with an autophagy activator (MG132) or inhibitors (wortmannin, bafilomycin A1) significantly increased and decreased the number of small ASB10-stained vesicles, respectively. No discernible differences in the colocalization of large ASB10-stained structures with ubiquitin or HDAC6 were observed between dermal fibroblasts derived from a normal individual and a patient with primary open-angle glaucoma carrying a synonymous ASB10 mutation.

**Conclusions:**

Our evidence suggests that ASB10 may play a role in ubiquitin-mediated degradation pathways in TM cells.

## Introduction

Primary open-angle glaucoma (POAG), a degenerative optic neuropathy, is a common cause of irreversible blindness. POAG is frequently associated with elevated intraocular pressure (IOP) caused by a dysfunction in the aqueous humor outflow pathway in the anterior segment of the eye [1,2]. Trabecular meshwork (TM) cells are responsible for establishing and monitoring aqueous outflow resistance and maintaining IOP homeostasis. TM cells also function as phagocytic cells to clear aqueous humor of debris and cellular components before they cross the less porous juxtacanalicular region and drain into Schlemm’s canal.

At the molecular level, the pathogenesis of POAG is complex. Genetic linkage analysis has identified at least 20 chromosomal loci for POAG [3-6]. Our group recently identified the causative gene at the glaucoma 1, open angle, F (GLC1F) locus as ankyrin repeat and suppressor of cytokine signaling (SOCS)-box-containing protein-10 (*ASB10*) [7]. A synonymous T255T variant in ASB10 segregated with disease in a large Oregon family with POAG, in which the GLC1F locus was originally mapped [7,8]. Other *ASB10* mutations were subsequently identified in two independent American and German cohorts. Since this gene was originally identified in a family with POAG with elevated IOP, we hypothesized that *ASB10* expression in the TM may play a role in outflow resistance. Indeed, when we silenced the *ASB10* gene, there was increased resistance to aqueous outflow in perfused human anterior segments [7]. However, the mechanism by which *ASB10* affects outflow facility and the biologic function of the ASB10 protein remain unknown.

**Figure 1:**
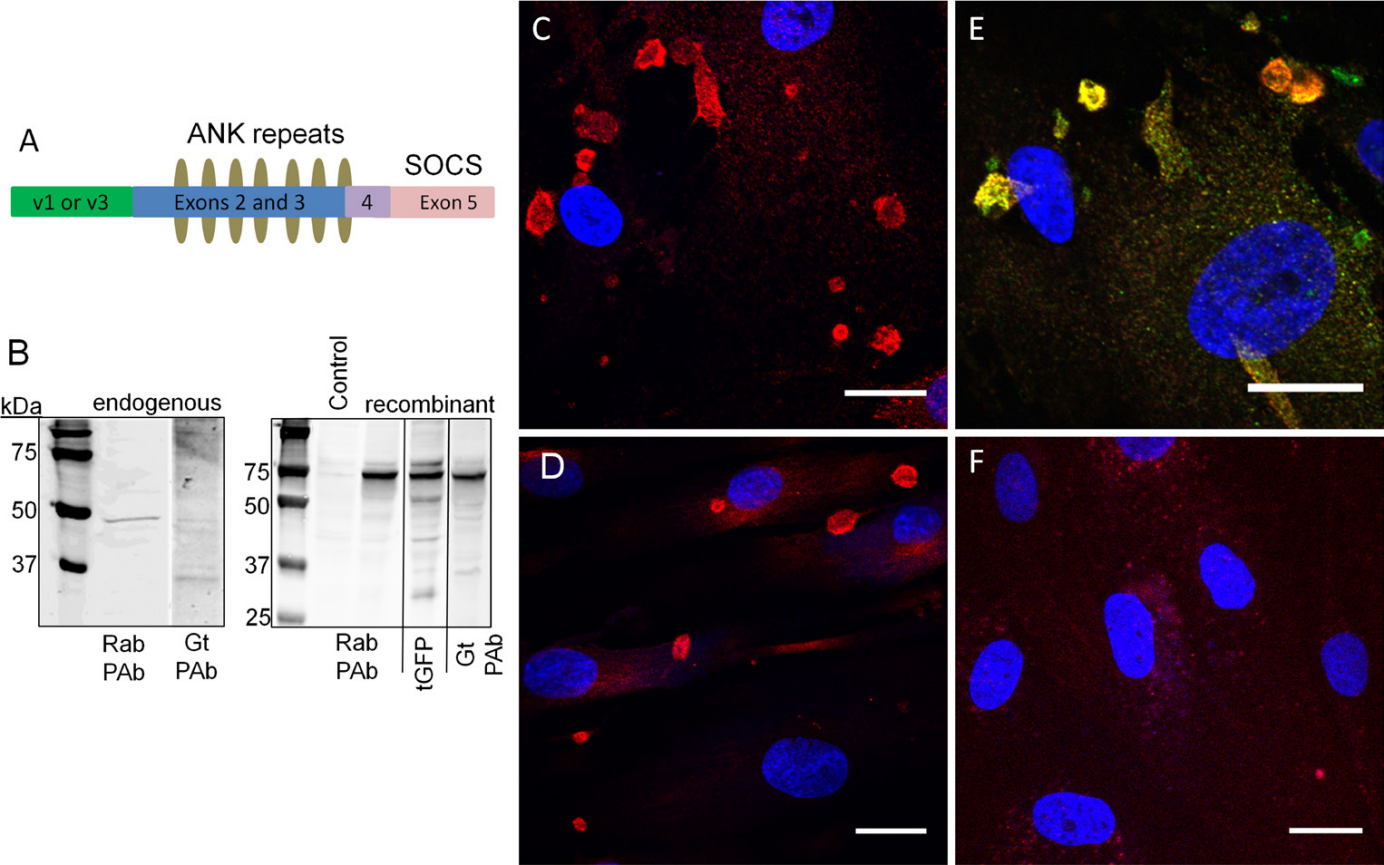
Characterization of ankyrin repeat and suppressor of cytokine signaling (SOCS) box containing protein-10 antibodies. **A**: A schematic diagram of ankyrin repeat and suppressor of cytokine signaling (SOCS) box containing protein-10 (ASB10) shows the position of the alternatively spliced N-terminus (variant 1 (v1) or variant 3 (v3); green), the ankyrin (ANK) repeats (olive ovals) and the SOCS box (pink). **B**: Western immunoblotting was performed to detect endogenous ASB10 in human trabecular meshwork (HTM) cell lysates (left panel) or 293 cells transfected with recombinant ASB10 variant 3 with a green fluorescent protein (GFP) tag at the C-terminus (right panel). The control was mock-transfected. Immunoblots were probed with the rabbit polyclonal ASB10 antibody (Rab PAb), the mouse monoclonal to turbo GFP (tGFP), or the goat polyclonal ASB10 antibody (Gt PAb). Molecular weight markers are shown in kDa. **C–F**: Immunofluorescence and confocal microscopy of HTM cells (**C, E, F**) and normal dermal fibroblasts (**D**) was performed using the rabbit polyclonal antibody (red, all images) and the goat polyclonal antibody (**E**, green). A negative control with no primary antibody is shown (**F**). Nuclei were stained with 4',6-diamidino-2-phenylindole dihydrochloride (DAPI). Scale bars=20 µm.

*ASB10* is one of 18 members of a family of *ASB* genes [9]. Members are related due to the presence of a varying number of ankyrin (ANK) repeat protein domains and a C-terminal suppressor of cytokine signaling (SOCS) box. The *ASB10* gene encodes seven ANK repeat domains and a SOCS box at the C-terminus (Figure 1A). Unique N-termini arise from alternative 5′ exon usage resulting in two ASB10 isoforms: variant 1 (v1) and variant 3 (v3). ANK repeats are one of the most common structural motifs and typically mediate specific protein–protein interactions [10,11]. The number and structure of ANK repeats are likely essential for defining which target substrate the ASB protein will bind. The SOCS box recruits the multisubunit E3 ubiquitin ligase complex, which then ubiquitinates the protein bound to the ANK repeats [12-14]. For instance, ASB3 and ASB9 mediate ubiquitination and degradation of tumor necrosis factor-alpha type II receptor and creatine kinase B, respectively [15,16], while ASB4 mediates insulin receptor substrate 4 degradation [17]. ASB family proteins can therefore play significant roles in ubiquitin-mediated degradation pathways and have been implicated as negative regulators of cellular signaling [15].

There are two main catabolic pathways for degrading cellular materials: the ubiquitin-proteasome system (UPS) and the autophagy-lysosomal (AL) pathways. These were originally thought to be distinct, but more recent studies indicate that under basal conditions, autophagy can participate in clearing ubiquitinated substrates [18]. Autophagy is a constitutive recycling process in which cargo destined for degradation is delivered to lysosomes in a step-wise process and is an essential process that maintains cellular and tissue homeostasis [19-23]. In macroautophagy, the main type of autophagy, a characteristic cup-shaped, double-membraned autophagosome encloses and sequesters cargo destined for degradation [23-26]. The autophagosome fuses with late endosomes to form amphisomes, which fuse with lysosomes to become autolysosomes [21]. Impaired lysosomal degradation in oxidatively stressed TM cells has been implicated in the pathogenesis of glaucoma [27].

Since other ASB proteins bind to and ubiquitinate specific cellular substrates for degradation, we hypothesized that ASB10 may serve a similar function in TM cells. As the first step to explore the biologic function of ASB10, we evaluated endogenous ASB10 expression in cultured TM cells and colocalized ASB10 antibodies with various biomarkers of the UPS and AL degradation pathways.

## Methods

### Primary cell culture

Primary human TM (HTM) cells were isolated and cultured as described previously [28,29]. Briefly, TM tissue was dissected from human donor eyes acquired from Lions Eye Bank (Portland, OR). Use of human cells and tissue was approved by the Oregon Health & Science University Institutional Review Board, and experiments were conducted in accordance with the tenets of the Declaration of Helsinki. HTM cells from four individuals were evaluated (average age=25 years; range=4–49 years). Results shown were consistent among all four cell lines used. HTM cells were cultured in medium-glucose Dulbecco’s Modified Eagle’s Medium (DMEM; Invitrogen, Carlsbad, CA) containing 10% fetal bovine serum (FBS) and 1% penicillin-streptomycin-gentamicin [30]. Primary HTM cells were used until passage 6.

Dermal fibroblasts were grown from punch skin biopsies from two of the GLC1F family members and an unrelated individual. One family member was diagnosed with POAG, while the other had no clinical signs of glaucoma noted during routine eye examinations. Informed consent was obtained from each individual. Skin biopsies were placed in flasks containing low glucose DMEM supplemented with 10% FBS and 1% penicillin-streptomycin. In addition, normal adult human dermal fibroblasts were obtained from ATCC (Manassas, VA) as an additional unrelated control. The fibroblasts were grown in fibroblast basal medium supplemented with the low serum fibroblast growth kit (ATCC). DNA sequencing confirmed that these fibroblasts were derived from an individual without the T255T synonymous mutation and had no other ASB10 exonic mutations. Dermal fibroblasts were used up to passage 8.

### Antibodies

Two polyclonal antibodies against ASB10 were used: a rabbit polyclonal (Sigma Aldrich, St. Louis, MO) and a goat polyclonal (Santa Cruz Biotechnology, Santa Cruz, CA). The rabbit polyclonal recognizes ASB10 ankyrin repeats 2 to 5 (exons 2 and 3), and the goat polyclonal recognizes an internal region of ASB10. The following antibodies were also used: mouse monoclonal ubiquitin and K48-ubiquitin (05–1307) and K63-ubiquitin (05–1308) rabbit monoclonal antibodies (EMD Millipore, Billerica, MA); mouse monoclonal alpha4 subunit of 20S proteasome and mouse monoclonal HSP70 antibodies (ENZO Life Sciences, Farmingdale, NY); mouse monoclonal antibodies HDAC6, LAMP1, and Rab7 (Abcam, Cambridge, MA); and mouse monoclonal antibodies p62 and LC3 (MBL International, Woburn, MA).

### Western immunoblotting

Radioimmunoprecipitation assay (RIPA) buffer (150 mM NaCl, 50 mM Tris, pH 7.2, 0.5% sodium deoxycholate, 1% NP-40, and 0.1% sodium dodecyl sulfate) containing protease inhibitor cocktail (Sigma) was added to flasks of confluent HTM cells. Proteins in cell lysates were separated on 10% sodium dodecyl sulfate–polyacrylamide gel electrophoresis gels (BioRad Labs, Hercules, CA) under reducing conditions and transferred to nitrocellulose. Immunoblots were probed with rabbit or goat anti-ASB10 polyclonal antibodies. Secondary antibodies were IRDye 700–conjugated antirabbit and IRDye 800-conjugated antigoat (Rockland Immunochemicals, Gilbertsville, PA). Immunoblots were imaged using the Odyssey infrared imaging system (Li-Cor Biosciences, Lincoln, NE).

### Recombinant ankyrin repeat and suppressor of cytokine signaling box containing protein-10 and immunoprecipitation

To generate recombinant ASB10, transfection-ready ASB10 variant 3 (rASB10v3) DNA in the pCMV6-AC vector containing a C-terminal green fluorescent protein (GFP) tag was purchased from Origene Technologies (Rockville, MD). Eight µg of plasmid DNA was transfected into 293 cells using Lipofectamine, and RIPA lysates were harvested 48 h later. Control lysates were prepared from a mock-transfection. Immunoblots were run as above and probed with rabbit or goat anti-ASB10 antibodies and mouse antiturbo GFP antibody (Origene).

For the immunoprecipitation experiments, HTM cells were transfected with 4 µg rASB10v3 with Amaxa nucleofection and grown for 3 days. Cells were washed with phosphate buffered solution (PBS; 150 mM NaCl, 2.7 mM Na_2_HPO_4_, pH 7.2), lysed, and immunoprecipitated with the Pierce Crosslink Immunoprecipitation kit (Thermo Fisher Scientific, Rockford, IL) and the mouse antiturbo GFP antibody (Origene). Protein complexes were separated on 4%–12% gels, and immunoblots were probed with antiubiquitin or anti-HSP70. Western immunoblots were imaged on the Odyssey as above.

### Generation of ankyrin repeat and suppressor of cytokine signaling box containing protein-10-silencing lentivirus

Silencing lentivirus that targets all ASB10 transcripts (sh1; exon 2) and control short, hairpin ribonucleic acid (shRNA) (shCtrl) that does not target any known transcript were designed and prepared as described previously [7,31]. For this study, we also designed a second shRNA that targets exons 5–6 (sh177). The sequence of the sh177 shRNA is as follows: 5′-CAC CGC TCT ACT AGA TGT CCA TGG CCG AAG CCA TGG ACA TCT AGT AGA GC-3′.

Silencing lentivirus was generated in 293FT cells with ViraPower packaging mix (Invitrogen) following the manufacturer's instructions [7,31]. To analyze the efficacy of messenger ribonucleic acid (mRNA) and protein knockdown in TM cells, shRNA silencing lentivirus (10^6^ pfu) was added with 6 µg/ml polybrene (Sigma, St. Louis, MO) at the time of plating in serum-containing medium. HTM cells were grown for 3 days to allow infection and silencing. RNA was isolated, and quantitative PCR (qRT-PCR) was performed using the DyNAmo HS SYBR Green qPCR kit (Thermo Sci., Lafayette, CO) and a Chromo4-equipped thermocycler (Biorad) [32]. ASB10 mRNA levels were normalized for 18S RNA, which acted as a housekeeping gene. To ensure knockdown of the ASB10 protein, western immunoblots of shRNA-silenced RIPA lysates were performed using the rabbit polyclonal antibody as above. Human anterior segment perfusion culture was set up and performed exactly as described for the sh1 ASB10-silencing lentivirus [7].

### Immunofluorescence and microscopy methods

HTM cells were grown on collagen-coated Bioflex membranes (FlexCell Int, Hillsborough, NC), which provides a more compliant surface for TM cells than glass or plastic, for 3 days [30,33]. Cells were then fixed with 4% paraformaldehyde (PFA) and blocked with CAS universal blocking reagent (Invitrogen). Membranes were removed from the dish and immunostained. Secondary antibodies were Alexa Fluor 488-conjugated antimouse or antigoat and Alexa Fluor 594-conjugated antirabbit or antigoat (Molecular Probes, Eugene, OR). Coverslips were mounted in ProLong gold containing 4',6-diamidino-2-phenylindole dihydrochloride nuclear stain (Invitrogen). Images were obtained with a Fluoview laser scanning confocal microscope (Olympus, San Diego, CA) and processed with FIJI software.

For super-resolution structured illumination microscopy (SIM), cells were grown and immunostained as described above. However, following the secondary antibody application and final wash step, antibodies were post-fixed with 4% PFA, and #1.5 coverslips were used. Images were acquired using a Zeiss (Thornwood, NY) ELYRA PS.1 system that reconstructs super-resolution images from a series of images acquired under spatially structured illumination [34,35]. Images were processed for SIM reconstruction using Zen 2010D software (Zeiss), and selected Z-planes were exported as TIFF images.

To analyze the area of ASB10-stained structures, the freehand selection tool in FIJI software was used to manually demarcate the circumference of each structure in a field. This was repeated for 25 separate confocal images from HTM cells derived from four individuals. The area of each structure was then calculated using the measure function. To analyze the number of ASB10-stained structures, immunofluorescence fields were imaged on a Leica DM500 fluorescence microscope with an attached digital camera and IM50 software. From the acquired images, the number of ASB10-stained large structures, that is, structures >5 µm in diameter, and the number of 4',6-diamidino-2-phenylindole dihydrochloride–stained nuclei were counted per field. At least 24 fields were counted for each treatment. The number of large structures per field was then divided by the number of nuclei, and an average number of large structures per nuclei per field±standard error of the mean (SEM) was calculated. To determine significance, analysis of variance (ANOVA) was used to compare ASB10-silenced or treated cells to control cells. p<0.05 was determined to be significant. In addition, Poisson probability distributions, which are used to describe the distribution of events with random occurrences, were calculated.

Imaris Bitplane software was used to provide a quantitative measurement of the colocalization of ASB10 with all the UPS and AL biomarkers used. Pearson’s correlation coefficient is a quantitative measurement that estimates the degree of overlap between fluorescence signals obtained in two channels [36]. Differences in the intensities of the signals are ignored, but similarity between shapes is considered. The Pearson’s coefficient values range from 1.0, an indication of complete colocalization of two structures, 0, which indicates no significant correlation, to −1, which indicates complete separation of two signals [36,37]. A Pearson’s correlation coefficient was calculated for each antibody pairing from raw compressed confocal z-stacks acquired using sequential scanning. Background correction values were identical for all images. A small box was drawn around the region of interest—either a large ASB-stained structure or small vesicles—using the square tool. Pearson’s correlation coefficients were then calculated by the software. The Pearson coefficients were calculated from multiple images. The Pearson coefficients were averaged, and a standard error of the mean was calculated. The degree of colocalization from the Pearson’s coefficient values was categorized as very strong (0.85–1.0), strong (0.49–0.84), moderate (0.1–0.48), weak (−0.26 to 0.09), and very weak (−1 to −0.27) based on a previously published description [37].

### Autophagy inhibition and activation assays

Pharmacological manipulators of autophagy were added to the HTM cells in culture. The HTM cells were plated and grown for 36 h before 5 µM MG132 (Enzo Life Sciences), 10 µM wortmannin, or 1 µM bafilomycin A1 (both Sigma) was added for an additional 18 h. MG132 inhibits proteasome degradation and activates autophagy, while wortmannin inhibits phosphatidylinositol 3-kinase (PI3K) and thus autophagosome formation and autophagy [38-40]. Neither agent is entirely specific to the UPS and AL pathways. Control cells were treated with an equal volume of vehicle (dimethyl sulfoxide). At the end of treatment, the TM cells were immunostained for confocal microscopy, and the number of ASB10-stained structures was counted as described above. For the outflow experiments, MG132 or vehicle control was applied at time point 0, and outflow was monitored for a further 43 h. Data for each eye were normalized to the average flow rate before treatment, and then data from individual eyes were averaged and the SEM was calculated. Data were analyzed with ANOVA where p<0.05 was considered significant.

## Results

### Characterization of ankyrin repeat and suppressor of cytokine signaling box containing protein-10 antibodies

First, we characterized two commercially available ASB10 antibodies. The epitope of the rabbit polyclonal antibody is from ANK2 to ANK5, while the goat polyclonal recognizes an internal region of ASB10. Both antibodies recognize the predicted molecular size of endogenous ASB10 (48 kDa) in immunoblots of RIPA lysates of HTM cells (Figure 1B). However, the goat polyclonal also recognizes other bands, which could represent alternative splice forms, degradation products, or non-specific bands. Both antibodies recognize recombinant ASB10 variant 3 (rASB10v3) from transfected 293 cells. Since rASB10v3 has a C-terminal GFP tag, the predicted molecular weight was larger, approximately 72 kDa. Again, the goat antibody had less apparent specific cross-reactivity than the rabbit polyclonal antibody. Next, we immunostained primary HTM cells (Figure 1C) and adult human dermal fibroblasts (Figure 1D) with anti-ASB10. Confocal microscopy showed that ASB10 was found in intracellular structures of varying sizes in both cell types. Some structures were perinuclear, but many were not. The rabbit (red) and goat (green) polyclonal antibodies colocalized at the same structures in HTM cells (Figure 1E). A negative control of the TM cells with no primary antibody is shown for comparison (Figure 1F). For the majority of the experiments presented, the rabbit antibody was used, and the goat polyclonal was used only when antibody combinations did not allow us to use the rabbit anti-ASB10.

**Figure 2:**
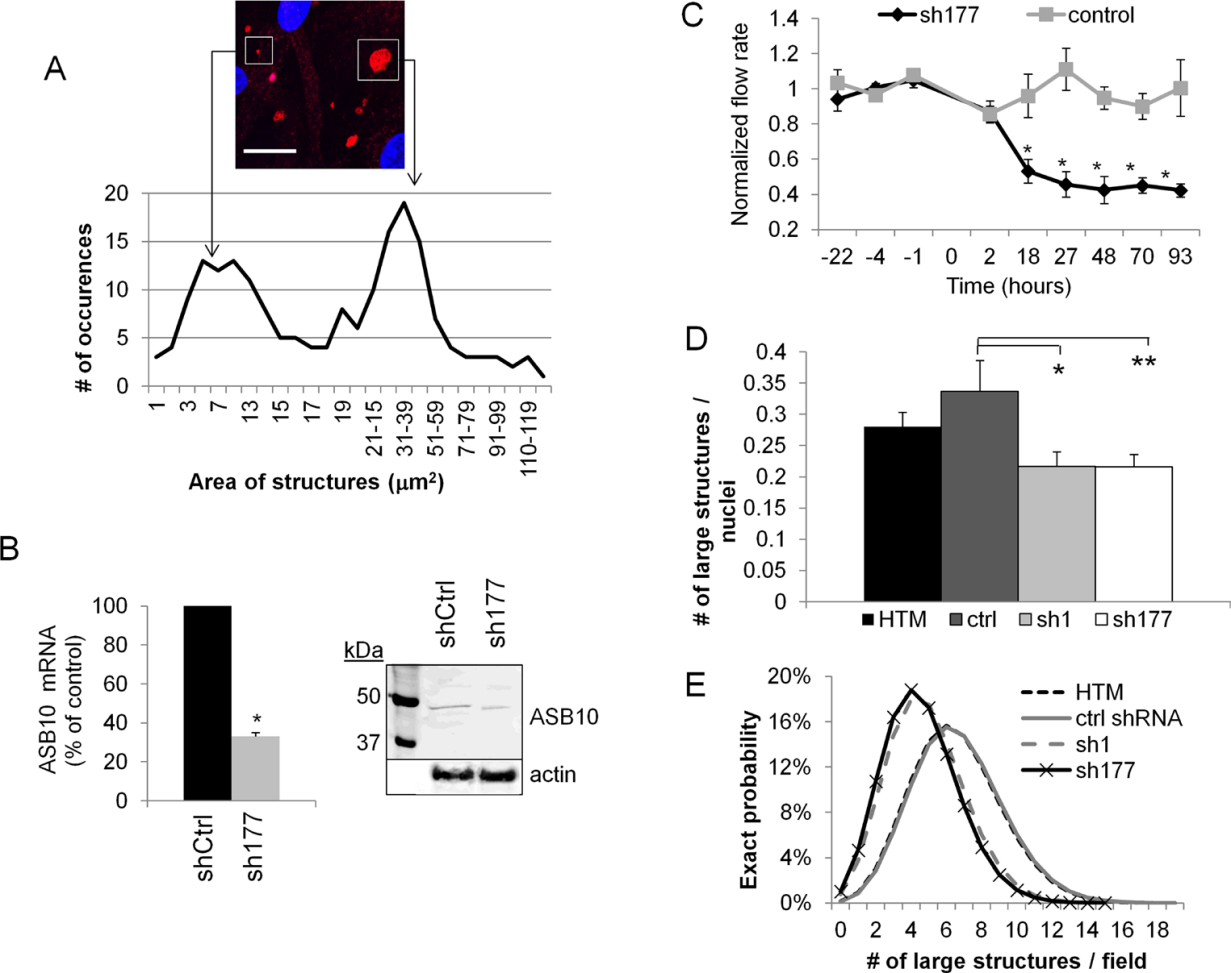
Characterization of ankyrin repeat and suppressor of cytokine signaling (SOCS) box containing protein-10-stained structures. **A**: The area of ankyrin repeat and suppressor of cytokine signaling (SOCS) box containing protein-10 (ASB10)-stained structures was measured using FIJI software from 25 confocal images of human trabecular meshwork (HTM) cells derived from four individuals. One microscope field shows examples of a large structure and small vesicles. Scale bar=20 µm. **B**: Efficacy of ASB10 knockdown by sh177 lentivirus was determined. Quantitative PCR (qRT–PCR) was used to measure ASB10 mRNA levels in HTM cells infected with 10^6^ pfu of sh177 or shControl (shCtrl) lentivirus. Values were normalized for 18S RNA. Values are presented as a percentage of the control±SEM; n=3; * p=0.0001 with ANOVA. Western immunoblot analysis shows significant knockdown of the ASB10 protein in the HTM cells. **C**: The sh177 ASB10-silencing lentivirus was applied to human anterior segment perfusion culture at time point 0, and outflow rate was monitored for a further 93 h. There was a significant (*p<0.001) decrease in outflow rate compared to the control-infected eyes. Error bars are SEM. **D**: The number of large (>5 µm diameter) ASB10-stained structures per nuclei in the control HTM and ASB10-silenced HTM cells was counted. Error bars are the SEM. *, p=0.033 and **, p=0.03 compared to control short, hairpin ribonucleic acid (shRNA)-infected HTM cells with ANOVA. **E**: Poisson probability distribution of the large ASB10-stained structures in control and ASB10-silenced HTM cells is shown.

The area of the ASB10-stained structures was measured (Figure 2A). There were two size ranges: a population of smaller vesicles, with areas from approximately 1 to 14 µm^2^ (diameters, 2–4 µm), and a population of larger structures with approximate areas from 20 to 60 µm^2^ (diameters, 5–9 µm). The average area was 23.76±1.53 µm^2^ (range, 1–128 µm^2^). The small vesicles were mainly spherical, while the larger structures were more irregularly shaped and included spheres, ovals, and triangular-shaped structures, sometimes with ruffled edges.

The number of large structures (>5 µm diameter) in the normal and ASB10-silenced HTM cells was then counted. Two shRNA silencing lentiviruses were used: One targets exon 2 (sh1) of ASB10, while the other targets exon 5 (sh177). Efficacy of the sh1 ASB10-silencing lentivirus was shown previously [7]. Here, we show that sh177 significantly decreased ASB10 mRNA levels by 70% compared to the control-infected cells (Figure 2B). The ASB10 protein was also decreased in western immunoblots. The densitometry of the bands showed an approximate 35% reduction compared to the control-infected HTM cells. Moreover, this ASB10-silencing lentivirus significantly reduced outflow rate in human anterior segment perfusion culture by a similar amount and in a similar time frame as the sh1 silencing lentivirus (Figure 2C) [7]. This outflow rate reduction when using lentivirus to silence full-length transcripts is consistent with our hypothesis that loss of full-length ASB10 may contribute to IOP increases in patients with glaucoma [7]. ASB10 silencing by two constructs targeting two regions of the gene strengthens the contention that the outflow effect is not due to mis-targeting or non-specific downstream effects of the shRNA.

The sh1 and sh177 ASB10-silencing lentiviruses were then incubated with HTM cells. There was no significant difference between the number of cell nuclei per field in sh1 or sh177 ASB10-silenced cells and the control-infected cells (data not shown). However, there was a significant decrease in the number of large structures present in ASB10-silenced cells for sh1 and sh177 (p<0.035; Figure 2D). The average number of large structures (>5 µm diameter) per nuclei was 0.336, 0.216, and 0.215 for the control-infected, sh1-silenced, and sh177-silenced cells, respectively. The number of ASB10-stained structures was also analyzed with the Poisson probability distribution function (Figure 2E). The number of large structures per field was plotted against the proportion of fields showing the number of structures. The distribution for both silenced groups was narrower and shifted to a lower number of structures per field compared to the control groups, which exhibited a wider distribution curve with a higher probability of large structures per field. Together, these data show that the number of large ASB10-stained structures decreases concomitantly as the ASB10 protein levels decrease.

### Ankyrin repeat and suppressor of cytokine signaling box containing protein-10 and the ubiquitin-proteasomal pathway

**Figure 3:**
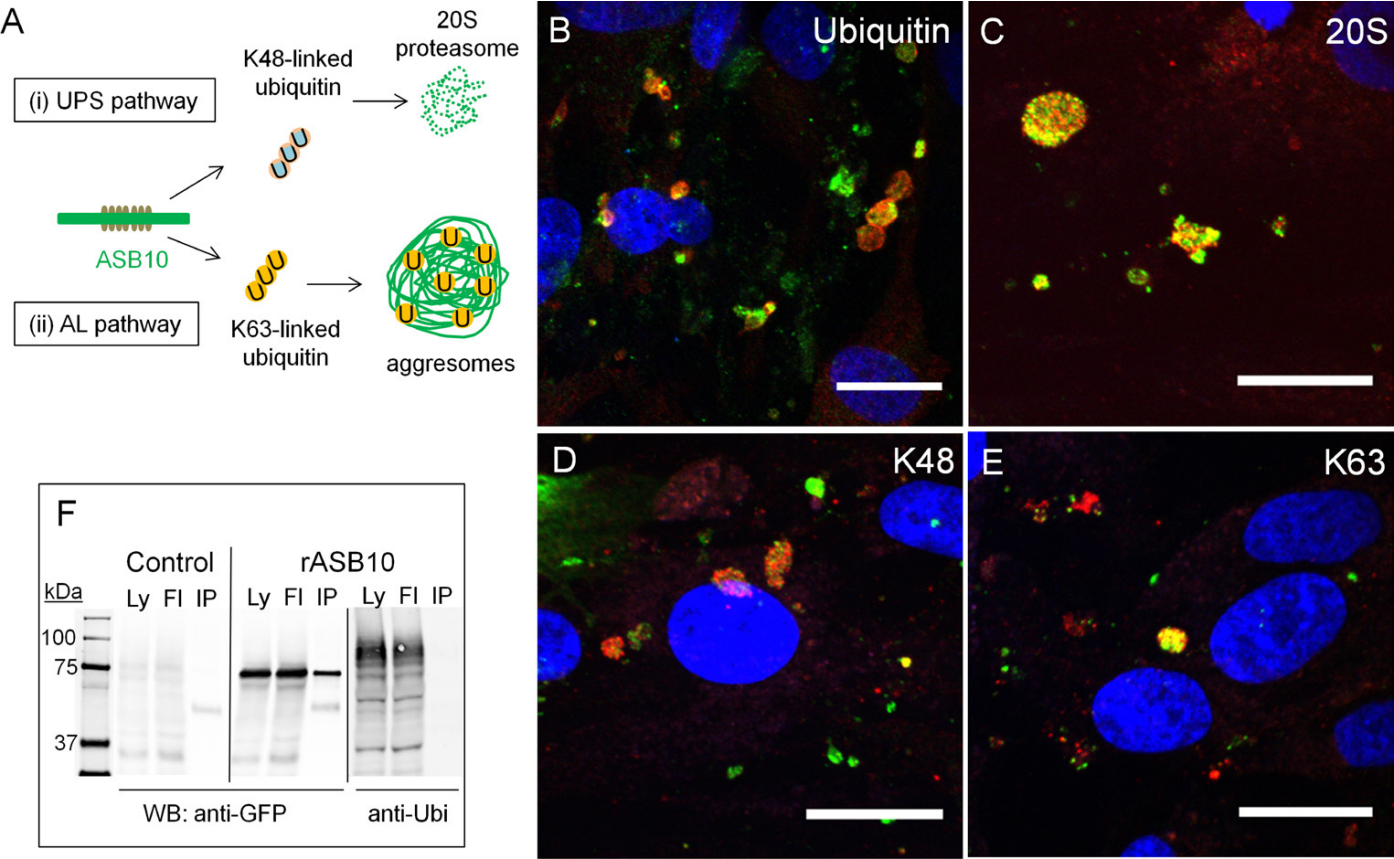
Ankyrin repeat and suppressor of cytokine signaling box containing protein-10 colocalizes with ubiquitin-mediated degradation pathway biomarkers in human trabecular meshwork cells. **A**: A schematic of ubiquitin-mediated degradation pathways are shown. **B–E**: Human trabecular meshwork (HTM) cells were immunostained with the rabbit polyclonal ankyrin repeat and suppressor of cytokine signaling (SOCS) box containing protein-10 (ASB10) antibody (red; **B**, **C**) and ubiquitin (green; **B**), the alpha 4 subunit of the 20S proteasome (green; **C**), the goat polyclonal ASB10 antibody (red; **D**, **E**), K48-linked ubiquitin (green; **D**), or K63-linked (K63; **E**) ubiquitin rabbit monoclonal antibodies. Nuclei were stained with 4',6-diamidino-2-phenylindole dihydrochloride (DAPI). Scale bars=20 µm. **F**: Coimmunoprecipitation of recombinant ASB10 variant 3 (rASB10) transfected into HTM cells are shown. rASB10 was immunoprecipitated using the green fluorescent protein (GFP) antibody. Western immunoblots show that ubiquitin (Ubi) is present in the cell lysates (Ly) and in the flow through (Fl), but not in the bound lane (IP), while rASB10 is present in all lanes, as detected with the GFP antibody. Untransfected HTM cells (control) show low levels of non-specific background immunostaining by the GFP antibody. Molecular weight markers in kDa are shown.

The SOCS box of ASB10 contains consensus sequence motifs for interaction with Cullin5 and elongin BC, which recruit proteins of the E3 ubiquitin ligase complex [14,15]. In other ASB proteins, the SOCS box recruits the ubiquitin complex, which then ubiquitinates the substrate bound to the ANK repeats suggesting a similar role for ASB10 [14-17]. Polyubiquitin chains are built via linkage at specific lysine (K) residues [41]. Proteins covalently tagged with polyubiquitin chains via K48 are destined for UPS degradation (Figure 3A(i)) [41]. Conversely, K63-linked ubiquitin is not typically associated with proteasomal degradation, but proteins tagged with K63 are degraded by the AL pathway (Figure 3A(ii)) [41-43]. Recently, linkage-specific antibodies have become available that can be used to distinguish polyubiquitin chains [44]. These linkage-specific antibodies are therefore powerful tools for interrogating protein modifications and providing clues to the function of endogenous proteins [45].

To examine ubiquitin colocalization with ASB10, we immunostained HTM cells with ubiquitin, ubiquitin K-linkage specific antibodies, and the alpha 4 subunit of the 20S proteasome (Figure 3). The ASB10-stained small vesicles and large structures showed some colocalization of ubiquitin (Figure 3B) and the 20S proteasome (Figure 3C). Using the K-linked ubiquitin antibodies, we detected colocalization with ASB10-stained structures, mostly with the larger structures rather than the smaller vesicles (Figure 3D,E). These immunostaining patterns suggest that a portion of ASB10 may be associated with the UPS and AL degradation pathways. However, in all cases, numerous vesicles contained ubiquitin, the K-linked antibodies, or 20S proteasome alone, which is not surprising since we would not expect full colocalization of these proteins with ASB10.

Next, we investigated whether ASB10 was ubiquitinated. Due to the low expression levels of endogenous ASB10, recombinant ASB10 was made by transfecting HTM cells with a GFP-tagged ASB10 construct. Recombinant ASB10 was immunoprecipitated using a GFP antibody, and immunoblots were probed with the ubiquitin antibody (Figure 3F). Ubiquitin was present in the cell lysates (Ly) and flow-through (Fl) on western immunoblots of the pull-down assays, but was not present in the bound rASB10 lane (IP). This suggests that ASB10 itself is not ubiquitinated.

### Ankyrin repeat and suppressor of cytokine signaling box containing protein-10 and autophagy-lysosomal pathway biomarkers

**Figure 4:**
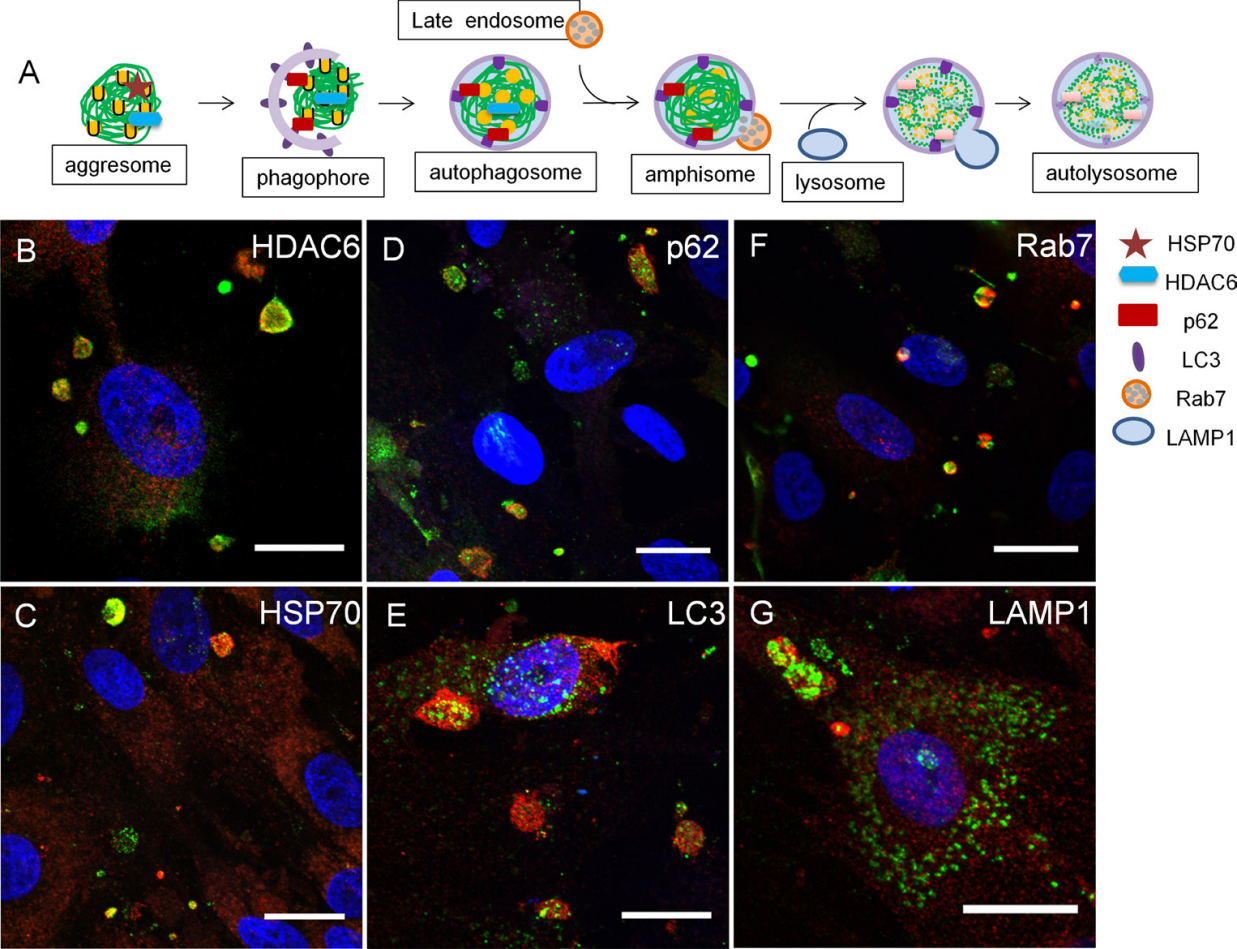
Ankyrin repeat and suppressor of cytokine signaling box containing protein-10 colocalizes with autophagy-lysosomal pathway biomarkers in human trabecular meshwork cells. **A**: A schematic shows the sequential steps and biomarkers of the autophagy-lysosomal (AL) pathway. **B–G**: HTM cells were immunostained with the rabbit ankyrin repeat and suppressor of cytokine signaling (SOCS) box containing protein-10 (ASB10) polyclonal antibody (red; **B–G**) and histone deacetylase 6 (HDAC6, green; **B**; aggresomes), heat shock protein 70 (HSP70, green; **C**; aggresomes), p62 (green; **D**, autophagosomes), light chain 3 (LC3, green; **E**, autophagosomes), Rab7 (green; **F**, late endosomes/amphisomes) or lysosomal-associated membrane protein 1 (LAMP1, green; **F**, lysosomes). Nuclei were stained with 4',6-diamidino-2-phenylindole dihydrochloride (DAPI). Scale bars=20 µm.

To further explore ASB10’s association with proteolytic degradation pathways, we colocalized ASB10 with various biomarkers of the AL pathway in the HTM cells. This pathway is characterized by the formation of characteristic cellular structures during the step-wise degradation process, each of which is associated with specific biomarkers (Figure 4A). Histone deacetylase 6 (HDAC6) and HSP70 are biomarkers of aggresomes, a preautophagic structure [20,46,47]. In addition, HDAC6 binds the C-terminus of ubiquitin via its zinc finger-binder of ubiquitin zinc finger domain [48,49]. In HTM cells, HDAC6 and HSP70 colocalized with ASB10 (Figure 4B,C). HDAC6 also immunostained some TM cell nuclei in a punctate pattern, consistent with HDAC6’s role as a histone deacetylase (data not shown).

Next, we investigated the expression pattern of the multifunctional scaffold protein, p62. This protein binds to K63-linked ubiquitin chains and is recruited into the double-membraned autophagosome [25,42]. p62 colocalized with large ASB10-stained structures, but small p62-stained vesicles in the cytosol were not colocalized (Figure 4D). Microtubule-associated protein 1 light chain 3 (LC3), a biomarker of autophagosomes, binds to p62 and is found as a cytosolic form (LC3-I) and an autophagosome-associating form (LC3-II) [50]. The antibody used in this study recognizes both forms of LC3. Clusters of LC3-stained vesicles were highly concentrated in the center of the large ASB10-stained structures (Figure 4E). LC3 was also found in single vesicles in a punctate pattern throughout the TM cell cytoplasm, which was not associated with ASB10.

We then immunostained HTM cells with the small GTPase Rab7. This is a biomarker of late endosomes within the endocytic pathway, but it can also associate with autophagosomes to form single-membraned amphisomes and mediate their fusion with lysosomes [51-53]. Not all Rab7 associated with ASB10, but that which did seemed to be predominantly located on one side of the ASB10-stained structures (Figure 4F). Finally, we examined immunostaining of LAMP1, an archetypal biomarker of lysosomes. LAMP1 staining was punctate in single vesicles in the cytoplasm of HTM cells, but clusters of LAMP1 vesicles were associated with ASB10-stained structures (Figure 4G). LAMP1 showed intense staining in the center of these structures.

### Super-resolution confocal microscopy

**Figure 5:**
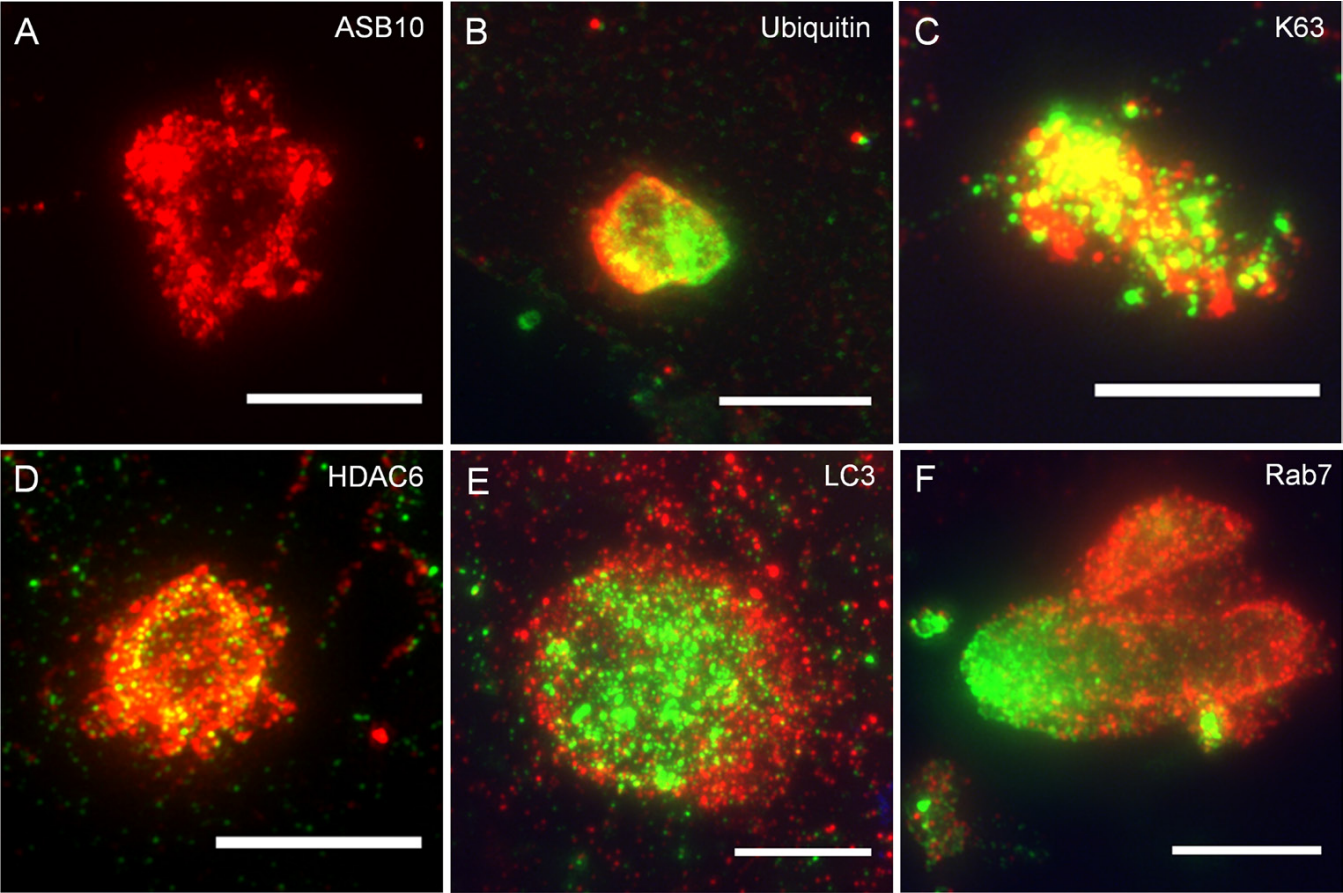
Super-resolution confocal microscopy of ankyrin repeat and suppressor of cytokine signaling box containing protein-10-stained structures colocalized with biomarkers of the ubiquitin-proteasome system and autophagy-lysosomal pathways. Human trabecular meshwork (HTM) cells were immunostained with the rabbit polyclonal ankyrin repeat and suppressor of cytokine signaling (SOCS) box containing protein-10 (ASB10) antibody (red; **A**, **B**, **D–F**), the goat polyclonal ASB10 antibody (red; **C**), total ubiquitin (green; **B**), K63-linked ubiquitin (green; **C**), histone deacetylase 6 (HDAC6, green; **D**), light chain 3 (LC3, green; **E**), and Rab7 (green; **F**). Scale bars=5 µm.

SIM is a new microscopy technique that provides substantially increased resolution (approximately 100 nm) compared to conventional confocal microscopy (to approximately 250 nm) [35]. This advanced light microscope methodology has several advantages over electron microscopy for identifying autophagosomes, including simpler immunostaining procedures for visualizing a larger number of cells. We therefore used SIM to further investigate ASB10-stained structures and the colocalization with selected UPS and AL biomarkers (Figure 5). Many of the large ASB10-stained structures appeared to be composed of clusters of smaller vesicles and appeared to form an empty sphere (Figure 5A). Smaller individual vesicles that were not clustered were also apparent (not shown). Since the higher resolution lends to more precise colocalization, SIM was also used to confirm the colocalization of ASB10 and a select number of UPS and AL biomarkers. Ubiquitin partially colocalized with ASB10, as if there was fusion of ASB10- and ubiquitin-stained structures (Figure 5B). This was also observed for the small vesicles. K63-linked ubiquitin chains were highly colocalized with ASB10 in large structures (Figure 5C). HDAC6 also colocalized with ASB10, and HDAC6 appeared as if was partially contained within an ASB10-stained sphere (Figure 5D). LC3-stained vesicles were clustered and surrounded by ASB10-stained structures, and there was partial colocalization (Figure 5E). Finally, Rab7 was associated with the ASB10-stained structures, but again the pattern suggested that there may have been fusion of the two structures (Figure 5F). Only a small amount of colocalization of Rab7 with ASB10 was observed. Together, this high-resolution microscopy data suggested that large ASB10-stained structures may form from clustering of smaller vesicles, and colocalization of ASB10 with K63-linked ubiquitin and HDAC6 was confirmed. Although ASB10 seemed to be located in the same structures with LC3 and Rab7, SIM showed that there was little colocalization.

### Quantitative analysis of ankyrin repeat and suppressor of cytokine signaling box containing protein-10 colocalization

**Figure 6:**
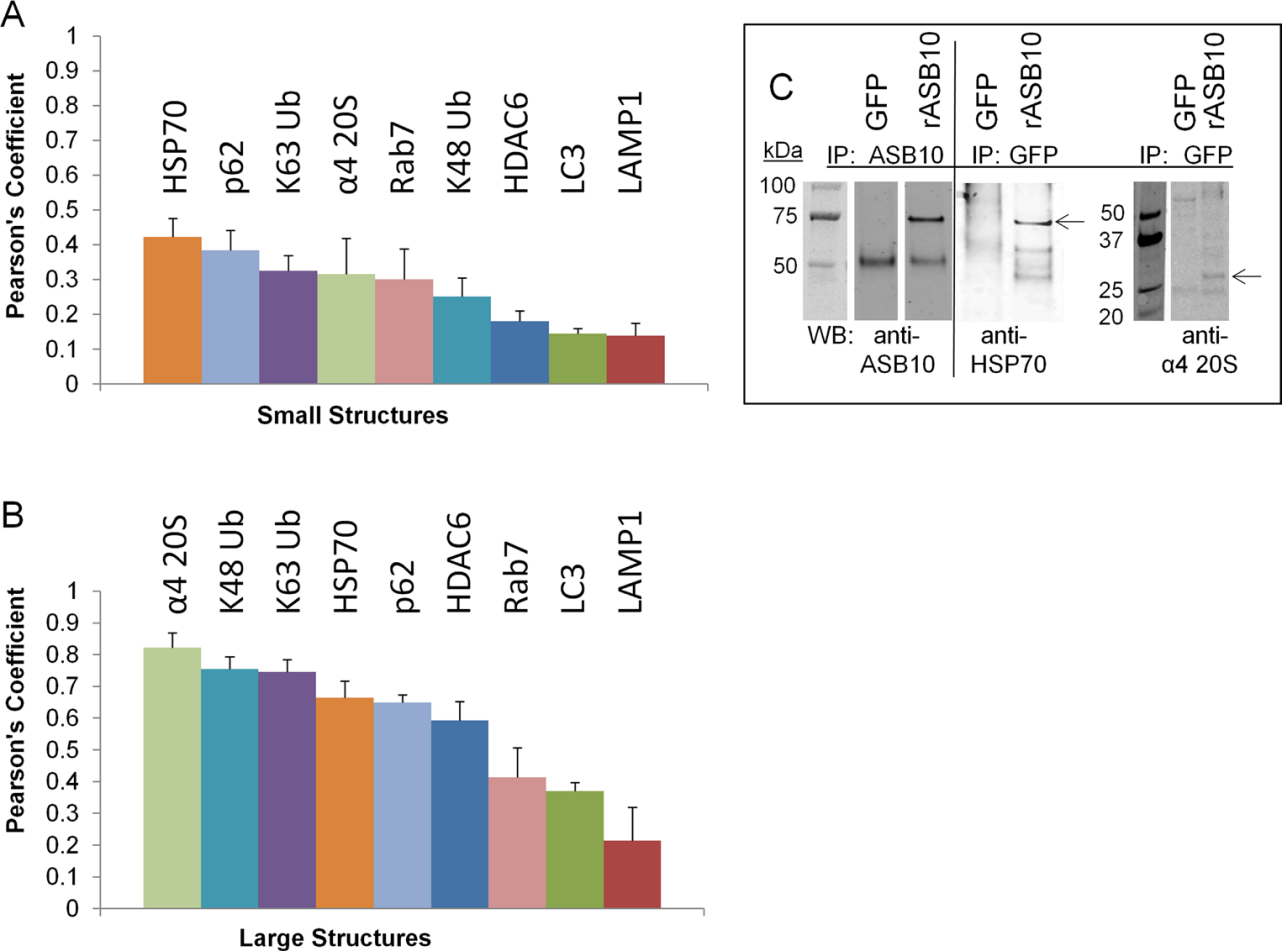
Quantitative analysis of ankyrin repeat and suppressor of cytokine signaling box containing protein-10 colocalization and coimmunoprecipitation. **A**, **B**: Pearson’s correlation coefficients were calculated using Imaris Bitplane software from compressed confocal z-stacks following background correction. The square box tool was used to demarcate large ankyrin repeat and suppressor of cytokine signaling (SOCS) box containing protein-10 (ASB10)-stained structures (**B**) and small vesicles (**A**). Error bars are the standard error of the mean. The number (*n*) of images analyzed for the small and large structures, respectively, was as follows: heat shock protein 70 (HSP70), n=3, 5; the α4 subunit of the 20S proteasome, n=2, 6; K63 ubiquitin, n=6, 6; K48 ubiquitin, n=7, 8; p62, n=9, 17; histone deacetylase 6 (HDAC6), n=3, 12; Rab7, n=3, 5; light chain 3 (LC3), n=10, 11; and lysosomal-associated membrane protein 1 (LAMP1), n=6, 3. **C**: Coimmunoprecipitation analysis of rASB10-green fluorescent protein (GFP) and GFP (control) was performed. Cell lysates were coimmunoprecipitated (IP) with GFP or ASB10 antibodies, and then the western immunoblot (WB) was probed with ASB10, HSP70, or the α4 subunit of the 20S proteasome antibodies. Lanes of the bound fractions are shown. The arrows point to the expected size of HSP70 and the α4 subunit of 20S proteasome. Molecular weights are shown in kDa.

To further analyze the colocalization of ASB10 with the UPS and AL biomarkers, we used Imaris Bitplane software to calculate the Pearson correlation coefficients. This quantitative measurement estimates the degree of overlap between fluorescence signals obtained in two channels [36]. Higher Pearson’s values represent a higher degree of colocalization of two signals. The degree of colocalization from the Pearson’s values were categorized as very strong (0.85–1.0), strong (0.49–0.84), moderate (0.1–0.48), weak (−0.26 to 0.09), and very weak (−1 to −0.27) as described previously [37]. Representative large structures and small vesicles were selected from compressed z-stacks by drawing a box around them. The software then calculated a Pearson’s coefficient for each combination of antibodies (Figure 6). Quantitation of multiple images showed that small ASB10-stained vesicles had only moderate colocalization with the AL and UPS biomarkers as the Pearson’s coefficients were at 0.42 and below (Figure 6A). However, analysis of the large ASB10-stained structures showed that ASB10 was strongly colocalized with the α4 subunit of the 20S proteasome, K48 and K63-linked ubiquitin, p62, HSP70, and HDAC6 (Pearson’s range, 0.59–0.82; Figure 6B). There was only moderate colocalization of large ASB10-stained structures with Rab7, LC3, and LAMP1 (Pearson’s range, 0.21–0.41). Of the biomarkers tested, this analysis suggests that ASB10 is more closely associated with biomarkers of the UPS pathway and preautophagic structures of the AL pathway than with mature autophagosomes and lysosomal markers.

To verify the colocalization data, we performed coimmunoprecipitation experiments. Recombinant ASB10-GFP or GFP control constructs were transfected into HTM cells and immunoprecipitated with the ASB10 or GFP antibody (Figure 6C). Western immunoblotting showed that HSP70 (expected size, 70 kDa) was in the bound lysates of the pull-down assays of the cells transfected with rASB10, but not in the GFP control-transfected cells. Similarly, a band corresponding to the expected size (28 kDa) of the α subunit of the 20S proteasome was found in the bound lysate of cells transfected with rASB10, but not in the control cells. These coimmunoprecipitation data confirm an association of ASB10 with biomarkers of the UPS and AL pathways.

### Autophagy inhibitors and activators

**Figure 7:**
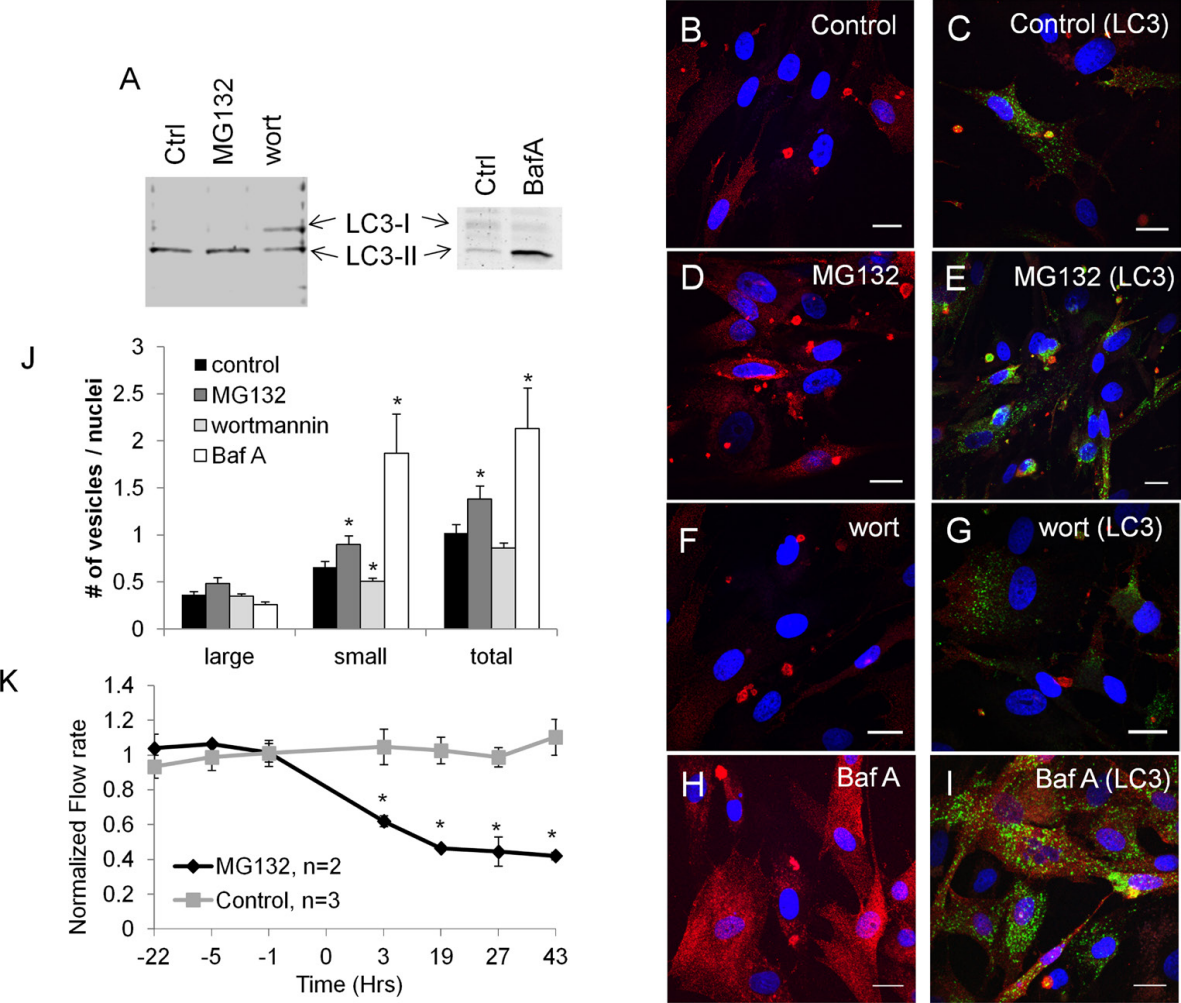
Autophagy assays. **A**: Western immunoblot with light chain 3 (LC3) antibodies was used to monitor autophagy in human trabecular meshwork (HTM) cells treated with vehicle control (ctrl), MG132 (5 µM), wortmannin (10 µM), or bafilomycin A1 (1 µM) for 18 h. **B–I**: Confocal microscopy was used to image HTM cells incubated with control (**B**, **C**), MG132 (**D**, **E**), wortmannin (**F**, **G**), and bafilomycin A1 (**H**, **I**). Representative images of immunostaining with the ankyrin repeat and suppressor of cytokine signaling (SOCS) box containing protein-10 (ASB10) antibody alone (red; **B**, **D**, **F**, **H**) or colocalized with LC3 (green; **C**, **E**, **G**, **I**). Nuclei were stained with 4',6-diamidino-2-phenylindole dihydrochloride (DAPI). Scale bars=20 µm. **J**: The number of ASB10-stained structures (large, small, and total) per nuclei was counted for each treatment. Note that for bafilomycin A1 the number of small vesicles was an approximation as there were too many to count accurately. Error bars are the SEM. *, p<0.05 with ANOVA. **K**: MG132 was applied to human anterior segment perfusion culture. A significant decrease in outflow rate was observed by 3 h after application compared to the vehicle control-treated eyes. Data are the average±SEM. * p<0.05 with ANOVA.

ASB10 immunostaining was analyzed in the presence of known autophagy inhibitors (wortmannin and bafilomycin A1) and an inhibitor of proteasomal degradation and activator of autophagy (MG132). First, conversion of endogenous LC3 from LC3-I to LC3-II was assessed to monitor autophagy (Figure 7) [38,39]. LC3-II is associated with autophagosomes. HTM cells were serum-starved and incubated with the pharmacological agents for 18 h. In the control and MG132-treated HTM cells, LC3-II was the most abundant form, whereas wortmannin partially inhibited LC3-I to LC3-II conversion (Figure 7A). Bafilomycin A1 caused an accumulation of LC3-II, as was expected. Next, we immunostained HTM cells with ASB10 (red) or LC3 (green) antibodies to monitor autophagy (Figure 7B–I). In the presence of MG132, which inhibits proteasomal degradation and activates autophagy, there was a significant increase in the number of ASB10-stained structures, especially small cytosolic vesicles, and a concomitant increase in LC3 immunostaining (Figure 7D,E). Other biomarkers (p62, LAMP1, Rab7) also seemed to increase (data not shown). Conversely, with the wortmannin treatment, which inhibits autophagosome formation, there was a slight decrease in the number of ASB10-stained structures, and there was a substantial decrease in the association of large ASB10 structures and LC3 (Figure 7F,G). Treatment with bafilomycin A1, which inhibits degradation of autophagic cargo inside autolysosomes, caused a large increase in punctate cytosolic ASB10 staining. However, there was no significant change in the number of larger ASB10-stained structures (Figure 7H,I). Bafilomycin A1 treatment caused a large increase in the number of LC3-stained vesicles, as expected. The number of ASB10-stained structures was counted for each treatment (Figure 7J). MG132 and bafilomycin A1 treatment increased the number of small vesicles compared to vehicle control, thus significantly increasing the total number of ASB10-stained structures. Wortmannin treatment significantly decreased the number of small vesicles, but there was no significant change in the large structures or in the overall number of ASB10-stained structures. In addition, we tested the effect of MG132 on outflow rate in human perfusion culture. Application of MG132 significantly decreased outflow rate compared to vehicle control within 3 h, and this decrease was maintained until the end of perfusion at 43 h (Figure 7K).

### Ankyrin repeat and suppressor of cytokine signaling box containing protein-10 immunolocalization in adult dermal fibroblasts

**Figure 8:**
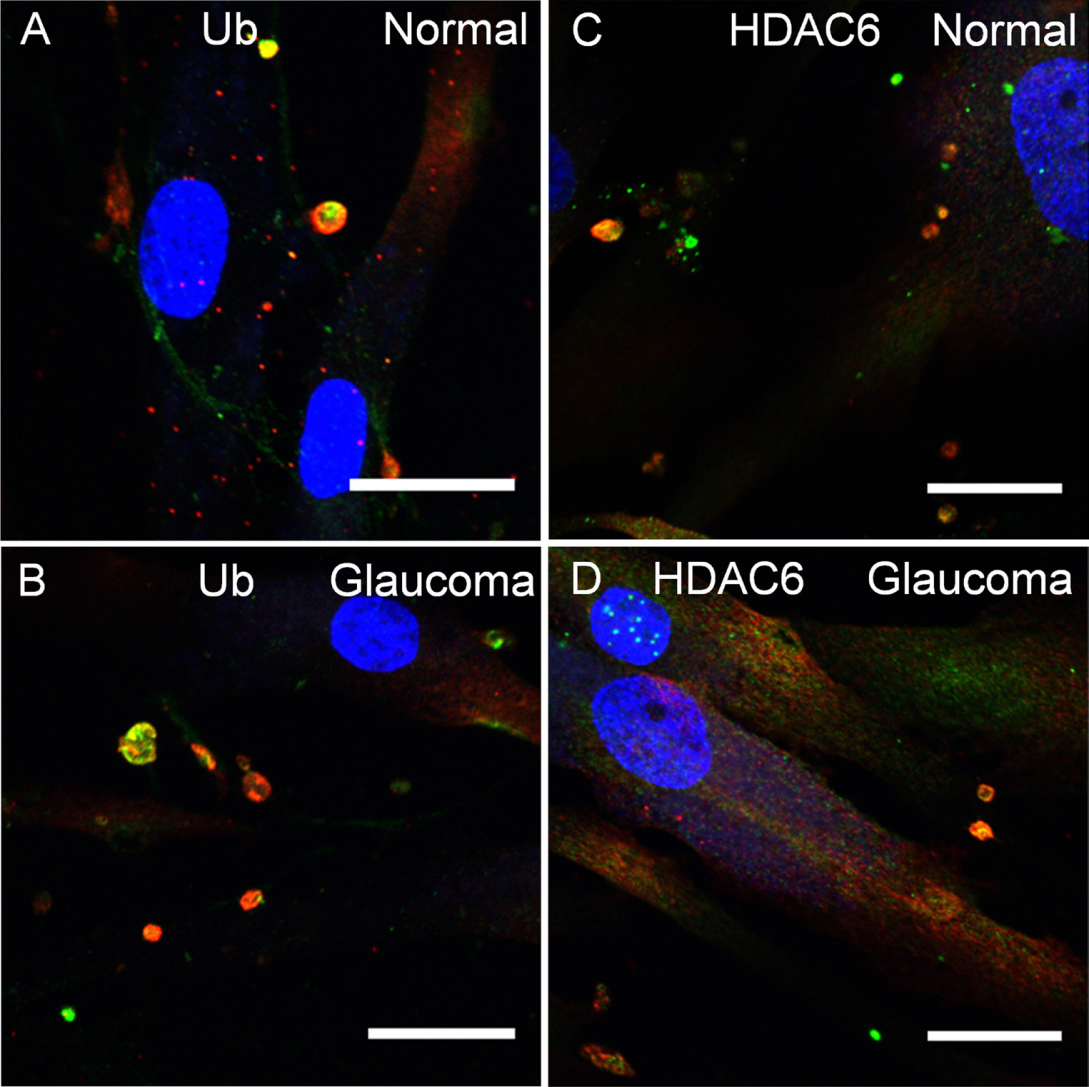
Ankyrin repeat and suppressor of cytokine signaling box containing protein-10 immunostaining of adult human dermal fibroblasts. Dermal fibroblasts were derived from normal subjects with no history of glaucoma (**A**, **C**) and a family member with glaucoma 1, open angle, F (GLC1F) with primary open-angle glaucoma (POAG) carrying the synonymous T255T mutation (**B**, **D**). Images show the colocalization of ankyrin repeat and suppressor of cytokine signaling (SOCS) box containing protein-10 (ASB10) (red) with ubiquitin (green, **A**, **B**) and histone deacetylase 6 (HDAC6, green, **C**, **D**). Nuclei were stained with 4',6-diamidino-2-phenylindole dihydrochloride (DAPI). Scale bars=20 µm.

Finally, we evaluated ASB10 immunostaining in adult dermal fibroblasts derived from skin biopsies from one affected and two non-affected members of the family with GLC1F POAG and an unrelated individual. Three normal cell lines and one POAG cell line, which carried the T255T synonymous mutation, were tested. ASB10 was again found in intracellular structures in both normal dermal fibroblasts (Figure 8A,C) and in fibroblasts from the patient with POAG (Figure 8B,D). Colocalization of large ASB10-stained structures with ubiquitin and HDAC6, and other UPS and AL biomarkers (data not shown), showed no discernible differences in the fibroblasts from the normal individuals and the patient with POAG.

## Discussion

The UPS and AL degradation pathways were originally thought to be distinct but are now generally believed to be reciprocal processes in which inhibition of one pathway can activate the other [18]. In this study, we showed that ASB10 partially colocalized and coimmunoprecipitated with biomarkers of the canonical UPS pathway and the AL degradation pathways. These observations provide strong evidence that ASB10 may function in ubiquitin-mediated degradation pathways.

There are three classes of autophagy: macroautophagy (which involves formation of a characteristic autophagosome), microautophagy, and chaperone-mediated autophagy [24]. Microautophagy involves the direct engulfing of a target by lysosomes, whereas chaperone-mediated autophagy clears cytosolic proteins containing the lysosome-targeting motif, KFERQ. Since ASB10 colocalized with biomarkers of autophagosomes and does not contain the KFERQ motif, we presume that ASB10 is associated with macroautophagy, hereafter referred to as autophagy. When autophagy was induced by the proteasomal inhibitor, MG132, there was a significant increase in the number of ASB10-stained structures. Conversely, treatment with wortmannin, which inhibits autophagosome formation, decreased the number of small ASB10-stained structures, and association with LC3 was essentially lost. HDAC6 and p62 bind to ubiquitylated substrates [42,43,48,49]. p62 also interacts with LC3 on autophagosomes, providing a physical link between polyubiquitinated substrates and autophagic degradation of inclusion bodies [42,43]. Thus, there might be two routes by which ASB10 enters the autophagic pathway—via HDAC6 or via p62 binding to LC3. Pharmacological manipulation of the AL pathway and association of ASB10 with multiple autophagic biomarkers indicate that endogenous ASB10 can associate with the AL pathway.

Based on the colocalization with HDAC6 and coimmunoprecipitation with HSP70, we hypothesize that ASB10 may initially be sequestered in aggresomal holding stations to await entrance to the AL pathway. Aggresomes are transient structures that are usually spherical, with a diameter of 1–3 µm [47]. In TM cells, there was a highly abundant population of small spherical ASB10-stained vesicles (2–4 µm in diameter). Thus, the size and shape of these small ASB10 vesicles, and their colocalization with the aggresomal biomarkers HDAC6 and HSP70, provide strong evidence that ASB10 is associated with aggresomes. However, many studies report that aggresomes are present in the perinuclear region and are coincident with the microtubule organizing center [47]. Although ASB10-stained structures were sometimes observed perinuclearly, they did not show a preference for any particular cytosolic location and were distributed throughout the cell in the TM cells and in the adult dermal fibroblasts. This observation, as well as the detection of a population of larger structures (5–9 µm in diameter), might argue against these ASB10-stained structures being aggresomes. However, our results indicate that small ASB10-stained vesicles may aggregate into the larger structures. This has been shown in dendritic cells where large poly-ubiquitinated protein aggregates, termed DALIS, are formed [54]. Small DALIS have the ability to move and form larger aggregates. DALIS are transient in nature, they do not localize to the microtubule organizing center, and they require proteasome activity to clear them [54]. DALIS have also been found in other cell types where the DALIS are termed ALIS (aggresome-like induced structures). They may function as protein storage compartments, which are cleared by autophagy [55-59]. We propose that the larger ASB10-stained structures in TM cells and dermal fibroblasts are ALIS and their transient nature may explain why not all cells contain these structures.

Rab7 endosomes fuse with autophagosomes to form amphisomes [60,61]. Formation of this hybrid organelle represents a compartment where the autophagic and endocytic pathways converge. Since ASB10 and Rab7 were predominantly associated with large structures, this subset of ASB10-stained structures may represent amphisomes. ASB10 did not strongly colocalize with small Rab7-positive vesicles, which likely represent vesicles of the endocytic pathway. Therefore, we hypothesize that ASB10 associates with Rab7 at the convergence point of the endocytic and autophagic pathways. However, more experiments with other members of the Rab family will be required to confirm this hypothesis.

In TM cells, a functional decrease in the cellular proteolytic machinery was observed in oxidatively stressed TM cells, including an increase in autophagic vacuole content and impaired lysosomal function [27,62]. The authors suggest that this may lead to alterations in the outflow pathway. In the present study, ASB10 colocalized with autophagy biomarkers in normal TM cells under basal culture conditions. Therefore, autophagy appears to be a ubiquitous process during regular TM cell homeostasis. Moreover, application of a pharmacological agent, MG132, which inhibits proteasomal degradation and therefore activates autophagy, decreased outflow rate providing evidence that manipulation of these cellular degradation pathways can influence outflow resistance.

Motifs in the SOCS box of ASB proteins recruit the ubiquitin ligase complex, which then transfers ubiquitin to a specific cellular substrate bound to the ANK repeats [12,13,15-17]. In this study, ubiquitin did not coimmunoprecipitate with ASB10 suggesting that ASB10 itself is not ubiquitinated. However, the colocalization of ASB10 with ubiquitin suggests a specific role for ASB10 in the degradation process. Since other ASB proteins bind to and degrade specific cellular targets, we hypothesize that ASB10 may serve a similar functional role in TM cells. We speculate that ASB10 binds a specific substrate(s) and recruits the E3 ubiquitin ligase complex to ubiquitinate the bound substrate, and this entire ubiquitinated complex is then targeted for degradation. ASB10 may remain bound to the substrate to “chaperone” it through the degradative process, and ASB10 may or may not also be degraded at the same time. In support of this hypothesis is that ASB10 is more strongly colocalized (higher Pearson’s coefficients) with preautophagic structures and biomarkers of the UPS pathway than with autophagosomes and lysosomes. Further studies are required to identify potential target substrates of ASB10 to confirm these hypotheses. Interestingly, many of the non-synonymous POAG mutations are in the ANK repeats and the SOCS box [7,63]. This may indicate that the interaction of ASB10 with its substrates and recruitment of the ubiquitin ligase complex may be compromised in some patients with glaucoma. Since the number and structure of ANK repeats define the target substrate to which the ASB protein is bound, it is unlikely that there is compensation by other ASB family members. However, further studies are necessary to support these arguments.

Despite the burgeoning abundance of manuscripts describing autophagy in numerous cell types, diseases, and aging, few studies have investigated autophagy in relation to glaucoma, and most of these studies have focused on retinal ganglion cells (RGCs) [64-68]. The ASB10 protein is expressed in the retina [7], which suggests that ASB10 may also associate with autophagy pathways in RGCs. Optineurin, one of the archetypal glaucoma-causing genes, is usually degraded via the proteasome pathway in RGCs [69-71]. Conversely, mutated or upregulated optineurin is degraded via the autophagic pathway [68]. Since ASB10 was colocalized with biomarkers of the UPS and AL degradation pathways and pharmacological manipulation appeared to alter levels, both degradation pathways may be used in tandem to clear specific proteins from RGCs and TM cells.

A recent paper questioned whether ASB10 is a glaucoma susceptibility gene [63]. Although a significant difference was found comparing the frequency of case-specific and control-specific non-synonymous ASB10 variants (p=0.03), this disappears after multiple testing is accounted for. Interestingly, four mutations identified in the study’s POAG population are not found in any of the control populations, including the 3,500 samples from the National Heart, Lung and Blood Institutes Exon Sequencing Project (NHLBI ESP) [63]. Yet, because the frequency of variants identified in the NHLBI ESP is similar to that found in the Iowa glaucoma population (2.53% compared to 2.34%), the authors ruled out ASB10 as a glaucoma susceptibility gene. However, this NHLBI ESP population was not examined for glaucoma. Thus, a proportion of the individuals identified with ASB10 variants may actually be affected. ASB10 is a highly polymorphic gene. Therefore, much higher numbers of cases and controls will be needed than those used by Fingert et al. to find a statistically significant value.

Analysis of dermal fibroblasts from a patient from the GLC1F family, who carries the T255T synonymous mutation, showed no difference in the colocalization of ASB10 with ubiquitin or HDAC6 compared to a normal family member. This suggests that, at least for this mutation and cell type and for the biomarkers studied, ASB10 association with either degradation pathway was not impaired. It may not be surprising that differences between glaucoma and normal fibroblasts were not detected since autophagic structures are transient. Moreover, only half of the ASB10 protein synthesized would be affected by the heterozygous T255T mutation. Further analysis with recombinant proteins carrying POAG mutations in TM cells with multiple biomarkers of autophagy is required to fully investigate the potential dysregulation of ASB10 mutants in the cellular degradation process.

In conclusion, we have provided evidence that endogenous ASB10 is associated with ubiquitin-mediated degradation pathways in TM cells in basal culture conditions. The search for new treatments for neurodegenerative diseases such as Parkinson disease, Alzheimer disease, Huntington’s disease, spinocerebellar ataxis, and amyotrophic lateral sclerosis is now focusing on the autophagy pathway as a potential therapy [72]. This study provides a new line of investigation for potential therapies for patients with POAG harboring ASB10 mutations.
